# Getting a Transcription Factor to Only One Nucleus Following Mitosis

**DOI:** 10.1371/journal.pbio.0060229

**Published:** 2008-09-16

**Authors:** Emily J Parnell, David J Stillman

## Abstract

The Ace2 transcription factor from budding yeast has both a regulated nuclear localization signal and a regulated nuclear export signal, and Ace2 phosphorylation by the Cbk1 kinase results in Ace2 accumulation in daughter cells but not mothers.

The growth and development of all eukaryotes depends on the process of asymmetric cell division, in which a single cell gives rise to two genetically identical cells that adopt distinct fates [1,2]. Asymmetry occurs when two cells are exposed to different environmental stimuli or when specific mRNAs or proteins (known as cell fate determinants) are segregated unequally. In recent years, studies in a variety of organisms have furthered our understanding of how these factors can cause two cells from the same division to experience different transcriptional outcomes, and thus take different paths.

One model system for the study of asymmetric gene expression is the budding yeast Saccharomyces cerevisiae. Asymmetry in S. cerevisiae is defined as the difference between a “mother” yeast cell and the smaller “daughter” cell that forms by budding from the original mother cell. One well-characterized example of this asymmetry is the mother cell–specific expression of the *HO* endonuclease gene that causes mother cells to switch mating type, a programmed genetic recombination that changes this dimorphic organism from one sex to the other [3]. *HO* is not expressed in daughter cells because the Ash1 transcriptional repressor is present in much higher amounts in daughter than in mother cells. The *ASH1* gene is transcribed during early M phase in both mother and daughter cells, but the *ASH1* mRNA is transported to the distal tip of the daughter cell during mitosis at a time when the cytoplasms of the two cells are still connected. The mRNA is then translated in the daughter cell, and the Ash1 protein binds to the *HO* promoter and represses its transcription. A second example of asymmetric segregation of proteins in yeast is the daughter-specific nuclear localization of the Ace2 transcriptional activator [4]. Ace2 directs expression of daughter-specific genes involved in separation of mother and daughter cells following mitosis. In this case, the Ace2 protein is transcribed and translated in both mother and daughter cells, but accumulates specifically in the daughter cell nucleus. Studies have demonstrated that the mechanisms controlling asymmetric localization of Ash1 and Ace2 are distinct and require different sets of protein factors. A new study in *PLoS Biology* by Mazanka et al. [5] sheds light on the factors involved in regulating this unequal segregation with a detailed look at the mechanism underlying Ace2 asymmetry.

## Ace2 and Swi5 Are Homologous Transcription Factors with Different Roles

The Ace2 transcription factor shares many similarities with a homologous zinc finger DNA-binding protein, Swi5 [6]. Since Ace2 is only present in daughter cells, it is intriguing that Swi5 is the primary activator of *HO*, the gene expressed only in mother cells. Both Ace2 and Swi5 are regulated by a series of cell cycle events that culminates in expression of their target genes in late M phase, before mitotic division, and early G1, the first gap when the cell produces proteins and grows in preparation for division. The *ACE2* and *SWI5* genes are transcribed in G2, the second gap, following genome replication (S phase), and the proteins remain in the cytoplasm until the end of mitosis, when they translocate and accumulate in the nucleus and activate expression of target genes. The specific timing of nuclear translocation appears to be achieved by a regulated nuclear localization signal (NLS) present in a region highly conserved between the two proteins. Phosphorylation of specific residues in the vicinity of the NLS in Swi5 and Ace2 is thought to mask the ability of the NLS to be recognized by nuclear import factors, thereby maintaining Swi5 and Ace2 in the cytoplasm [7,8]. At the onset of anaphase (when sister chromatids separate), the Cdc14 phosphatase is activated and likely dephosphorylates both Swi5 and Ace2, allowing the NLS to be recognized and nuclear entry and accumulation to occur [9]. In addition to their similarities in cell cycle regulation, the Ace2 and Swi5 proteins have several regions of amino acid similarity, including a 95% similar DNA binding domain. Ace2 and Swi5 recognize and bind to the same sequences with equal affinity in vitro [10].

Despite the similarity in the Ace2 and Swi5 DNA binding domains, these transcription factors regulate expression of different genes in vivo [11]. Ace2 primarily activates genes involved in cell separation, while Swi5 predominantly activates genes that direct cell cycle progression. The localization patterns of Ace2 and Swi5 are also distinct. Swi5 accumulates in both mother and daughter nuclei, whereas Ace2 accumulates specifically in daughter nuclei, although it transiently appears in mother cell nuclei [4,12]. In addition to its regulated NLS, Ace2 also has a regulated nuclear export signal (NES) not present in Swi5. This NES is one of a class of sequences recognized by the Crm1 nuclear export factor [13]. Mutations in Crm1 or inhibition of Crm1 by the drug leptomycin B result in nuclear localization of Ace2 in both mother and daughter, although there is much less Ace2 in these nuclei compared to native daughter cells [12,14]. This result demonstrates that the NES is critical for asymmetric distribution of Ace2. Crm1-dependent NESs are typically rich in leucine residues, and these leucines are required for export activity. However, the leucine residues in the Ace2 NES are dispensable for its function, suggesting that it defines a unique class of export sequences [13]. The Crm1–Ace2 association is independent of Ran-GTP [5], and thus differs significantly from previously described interactions of NES sequences with the Crm1 exportin. Thus Ace2 has both a regulated NLS and a regulated NES; mutations at both of these elements can result in a constitutively nuclear protein [6].

## The Cbk1 Kinase Associates with Ace2 and Modulates Its Function

An additional distinguishing characteristic of Ace2 is its association with the Cbk1 protein, a kinase responsible for the daughter-specific nuclear localization of Ace2. Racki et al. [15] provided the first links between Ace2 and Cbk1. They screened for suppressors of the aggregation phenotype caused by a *cbk1* mutation and identified mutations in Ace2, in what we now know to be the Ace2 NES. In a recent paper, they identified point mutations in the essential *CRM1* gene encoding the export factor that also suppress *cbk1* defects [14]. Cbk1 is a member of the RAM (Regulation of Ace2 activity and cellular Morphogenesis) signaling pathway. There are at least six members of the RAM network that regulate Ace2 function as well as polarized growth and formation of mating projections: *CBK1*, *MOB2*, *KIC1*, *HYM1*, *TAO3*, and *SOG2* [16]. Mutants of RAM network genes, including *CBK1*, have a defective cell separation phenotype similar to that observed in *ace2* strains, resulting from decreased expression of Ace2 target genes. RAM mutants also display defects in bud site selection, a round cell morphology indicative of inefficient apical growth during early bud morphogenesis, and a failure to form mating projections. The defects in morphogenesis caused by RAM network mutations are fully independent of Ace2 [17].

**Figure 1:**
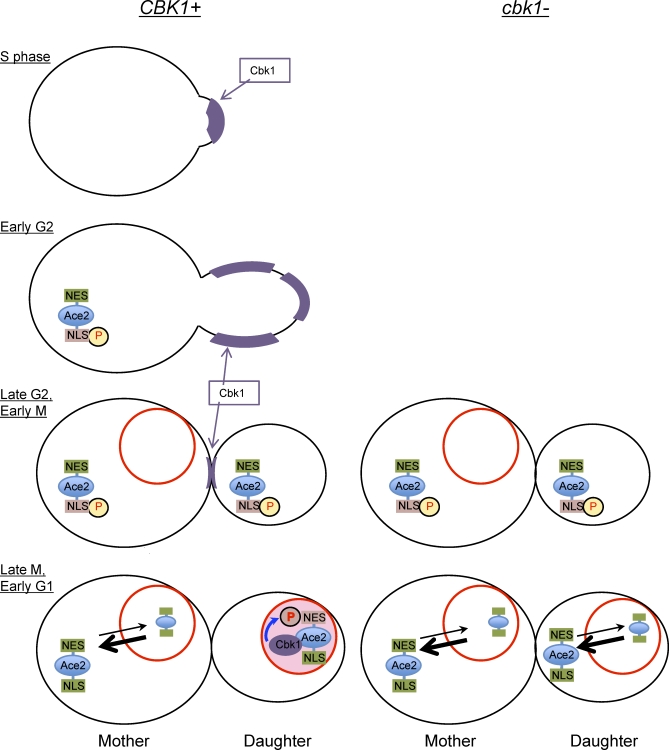
Ace2 Phosphorylation by Cbk1 Inactivates the NES and Allows Accumulation in the Daughter Cell Nucleus Localization of Cbk1 and Ace2 proteins at different phases of the cell cycle is shown in wild-type (left) and *cbk1* mutant (right) cells. Cbk1 protein (shown in purple) is found first at the cell cortex of the incipient bud in S phase, then at the surface of the growing bud, then at the bud neck, and finally enters the nucleus of daughter cells. Nuclear accumulation by Cbk1 and Ace2 are codependent. Ace2 protein is translated in G2 phase and is retained in the cytoplasm because phosphorylation masks its NLS. In late M phase, dephosphorylation activates the NLS and the protein enters the nucleus. In mother cells, nuclear localization is transient because the NES is dominant, driving Ace2 to accumulate in the cytoplasm. In daughter cells, phosphorylation by Cbk1 inactivates the NES, and Ace2 remains in the nucleus. In *cbk1* mutants, the Ace2 NES is active in mother and daughter cells, and the protein is exported from the nucleus; thus, a similar low level of Ace2 is seen transiently in both nuclei following cell division.

At the beginning of S phase, the Cbk1 protein is found at the cell cortex of the incipient bud and remains at the bud surface during bud growth. At the end of G2, Cbk1 moves to the bud neck and then translocates to the daughter cell nucleus in M phase (Figure 1) [12,15,17]. Ace2 is required for nuclear localization of Cbk1, suggesting that these proteins move to the daughter cell nucleus as a complex. In the absence of Cbk1, the daughter-specific nuclear localization of Ace2 is lost (Figure 1), and Ace2 target genes are no longer activated.

## Cbk1 Blocks Nuclear Export of Ace2 from the Daughter Cell Nucleus

Taken together, these observations have led to a model in which Cbk1 directs daughter-specific nuclear accumulation of Ace2 by blocking export of Ace2 from the daughter cell nucleus. It appears that in the mother cell, where Cbk1 is not detectable, the NES is dominant and Ace2 accumulates in the cytoplasm. Mazanka et al. [5] further elucidate the mechanism by which Cbk1 inactivates the NES in the daughter cell, and they have possibly identified an additional role for Cbk1 in regulating Ace2 function. Previous studies had demonstrated that Cbk1 phosphorylates Ace2 in vitro, but neither the specific residues phosphorylated nor the effects of these modifications were known [18]. Using peptide scanning arrays, Mazanka et al. [5] determined a consensus motif for the Cbk1 kinase and identified four potential target phosphorylation sites within Ace2. Importantly, two of these phosphorylation sites are within the NES of Ace2. Mutation of these residues to alanines caused Ace2 to weakly localize in both mother and daughter nuclei, similar to a *cbk1* phenotype. Ace2 protein that is unphosphorylated interacted with the Crm1 export factor in vitro, but this interaction was abolished either by phosphorylation of the two Cbk1 target sites in the Ace2 NES or by mutation of the phosphorylated serines to acidic amino acids. Thus, Ace2 interacts directly with Crm1, and phosphorylation by Cbk1 antagonizes this interaction, illustrating the mechanism by which Cbk1 blocks the export machinery. Mazanka et al. [5] have thereby presented the first direct evidence with purified components for inhibition of an NES by blocking association with export proteins, a mechanism distinct from other known classes of NESs, in which phosphorylation prevents export by facilitating recruitment of accessory factors or by relieving an inhibitory intramolecular interaction.

In contrast to a *cbk1* null mutant, the double alanine substitution mutant of Ace2 remained capable of expressing Ace2 target genes and promoting separation of mother–daughter pairs, albeit at a reduced level. Interestingly, combination of the two NES alanine mutations with an additional alanine mutation in a third Cbk1 consensus site distant from the NES further decreased both activation of Ace2 target genes and Ace2 binding to these promoters. These results uncover a possible additional function of Cbk1 in the regulation of Ace2, suggesting that Cbk1 phosphorylation is required for maximal transcriptional activity of Ace2. Thus, Cbk1 may serve at least two regulatory functions within Ace2; mutation of both is necessary to abrogate the expression of cell separation genes in daughters.

## How Is Daughter-Specific Localization Achieved?

In a series of elegant experiments, Mazanka et al. [5] also addressed exactly when Ace2 enters the daughter cell nucleus during the cell cycle. Using time lapse microscopy, they demonstrated that the Cbk1–Ace2 complex localizes to the daughter cell nucleus prior to cytokinesis. This result is quite surprising, as at this stage proteins are able to freely move between the mother and daughter cells, and suggests the existence of an active mechanism that restricts nuclear accumulation of the complex to daughter cells. In addition, they used a double mutant strain affecting both orientation of the mitotic spindle and checkpoint control, such that both nuclei frequently accumulate in one cell, either the mother or daughter. This experiment shows that the environment of the daughter cell is essential for nuclear accumulation of Ace2.

What is unique about the daughter cell that allows Ace2 to accumulate in the nucleus? Cbk1 localizes first at the tip of the incipient bud of the daughter cell, and subsequently moves to the bud neck (Figure 1). One might propose that the presence of Cbk1 in the daughter generates that unique environment necessary to promote nuclear entry of Ace2. However, Bidlingmaier et al. [17] observed that Cbk1 is present on both mother and daughter sides of the bud neck. Mazanka et al. [5] provide two possible models for restricting Ace2 nuclear entry to daughter cells: restriction of movement between mother and daughter by a barrier at the bud neck, or localization of proteins at the bud neck that inhibit formation of an active Cbk1 kinase except in daughter cells farther from the bud neck. The role of the other RAM network proteins in Ace2 regulation is poorly understood; perhaps one or multiple of these proteins plays a role in limiting Cbk1 activity to daughter cells.

The fact that the leptomycin B inhibitor of the Crm1 export factor differentially affects localization of Ace2 and Cbk1 raises other questions. Ace2 accumulates in both mother and daughter nuclei when export is blocked [12,14]. In contrast, leptomycin B treatment does not cause Cbk1 accumulation in mother cells [12,14]. Thus leptomycin B treatment results in Ace2, but not Cbk1, accumulation in mother cell nuclei. This result is consistent with the idea that the Cbk1 protein itself is restricted to daughter cells, providing a mechanism for daughter-specific transcriptional activation by Ace2.

However, one observation is not consistent with this attractive model. Bourens et al. [14] examined the *crm1* mutations that allow transcriptional activation by Ace2 in the absence of Cbk1, and found that these mutations result in both Ace2 and Cbk1 accumulation in the nucleus of mother cells. Thus there is a difference in Cbk1 localization depending on whether Crm1 activity is reduced by leptomycin B or by mutation. Ace2 is normally exported from the nucleus in G1 [6], and it is possible that in the *crm1* mutant the Ace2/Cbk1 proteins perdure in the daughter cell nucleus until this cell becomes a mother. Further work is needed on this question, and the study of other RAM pathway proteins and their functions may provide understanding of the subtleties of how asymmetric gene expression is achieved. Finally, it is intriguing that Cbk1 may be required for maximal DNA binding and transcriptional activation by Ace2, in addition to blocking Ace2 nuclear export, and further studies are necessary to determine how phosphorylation at a site outside the NES affects activation by Ace2.

In summary, Mazanka et al. [5] have elucidated several aspects of the mechanism by which the Cbk1 protein regulates asymmetric localization of the Ace2 transcription factor. Cbk1 is a member of the NDR/LATS kinase family, which is conserved from yeast to vertebrates. While the outcome of the phosphoregulation provided by members of this family appears to differ between organisms and proteins, dissection of the molecular details contributes to our understanding of the mechanisms that can be used to achieve asymmetry in gene expression following cellular division.

## Abbreviations

NES: nuclear export signal
NLS: nuclear localization signal
RAM: regulation of Ace2 activity and cellular morphogenesis
